# Organisational and Professional Integration Between Specialist and Primary Healthcare Services: A Municipal Perspective

**DOI:** 10.5334/ijic.5606

**Published:** 2021-05-24

**Authors:** Mona Jerndahl Fineide, Erna Haug, Catharina Bjørkquist

**Affiliations:** 1 Faculty of Health and Welfare, Østfold University College, Norway

**Keywords:** integration, coordination, organisational, professional, rainbow model

## Abstract

**Introduction:**

Care transitions between specialist and primary healthcare services for people with concurrent substance abuse and mental health problems are characterised by vulnerability and arbitrariness.

**Objectives:**

By studying factors that influence integration in a Norwegian context, this study aims to investigate, from a municipal perspective, why care transitions are still tricky after the introduction of the key Coordination Reform.

**Methods:**

This study has an explorative approach based on interviews with managers and front-line professionals in primary care. We applied the conceptual framework of functional and normative integration of the Rainbow Model.

**Results:**

The municipal actors emphasise that integration is hampered by limited cooperation with general practitioners in referrals to hospital, challenges of communication and loss of meeting points. They experienced close cooperation with sociomedical polyclinics for substance abuse, while challenges in cooperation with district psychiatric centres indicated an interdependence of functional and normative integration. Questioning hospital discharge of patients to primary care was a recurring theme for the municipal actors. Thus, the governing framework of the Coordination Reform has coexisted with fragmentation in organisational structures and divided professional cultures.

**Conclusions:**

The coexistence of the new and the old regimes seems to hamper functional and normative integration in care transitions.

## Introduction

People with concurrent substance abuse and mental health problems (dual diagnosis) are often in need of both specialist and primary healthcare services. This group has long been regarded as one of the most clinically complex [1,2]. Here, inter-organisational coordination is crucial in order to create integrated services. Coordination can be described as continuous processes where different parts or elements are inter-related, prioritised and adapted to each other and can be organised in different ways [3,4,5].

Recent decades have been an era of restructuring in Norwegian health and social services. The Escalation Plan for Mental Health 1998-2008 continued the deinstitutionalisation of mental health services in a shift from hospitals to primary care [6]. The 2002 Hospital Reform and the 2004 Substance Abuse Treatment Reform led to a reorganisation of specialist health services [7,8]. Despite several reforms aiming to improve challenges of fragmentation, coordination has remained a problem [9,10,11].

The Norwegian Coordination Reform of 2011 entitled “Proper treatment – at the Right Place and the Right Time” constitutes a new policy framework that can be seen as a common governance mechanism to secure inter-organisational integration between specialist and primary healthcare services. The key element in the reform was the transfer of responsibility from specialist to primary healthcare, thus requiring the establishment of new, more advanced services at local level [12,13]. The reform emphasised that stronger financial, legal and organisational measures should be taken to promote goals of coordinated services [14]. The reform also aimed at better integration and a more clearly defined role for general practitioners (GPs) in primary healthcare. GPs and municipalities both provide primary care, and GPs run private practices in contracts with municipalities [13,14,15]. An additional regulation at system level was the introduction of a national guideline for the assessment, treatment and follow-up of people with dual diagnosis. The guideline uses the term “integrated treatment” as an ideal for treatment where all measures and clinical support for people with dual diagnosis are combined within one treatment tradition or unit [16].

In 2015, the Norwegian Board of Health Supervision carried out a risk assessment of services for people with dual diagnosis. The report revealed that both hospital services and municipal health and social services were inadequate. The most serious problems were found to be lack of coordination between mental healthcare and interdisciplinary specialised treatment for substance use disorders. Examinations, assessment and diagnosis were reported as inadequate and municipal services had not been strengthened in line with the reduction in specialist services; municipal healthcare has only slightly increased its capacity and expertise after the introduction of the reform [17]. Recent parliamentary reports confirm breaches of legislation and shortcomings in municipal healthcare and district psychiatric centres (regional units under specialist healthcare) and poor inter-organisational coordination [18,19]. In sum, the number of hospital beds has fallen significantly. At the same time, there is increased pressure on primary care and an unexpected increase in the number of hospital admissions and readmission rates [20,21,22].

The policy framework of improved coordination, expressed through changed legal requirements, new funding schemes and official guidelines for admission and discharge of patients may be considered as a set of system-level management signals that could provide strong guidance on how to implement systems for organisational and professional integration. However, it is well known that implementation is demanding and that few of the ideas and tools that are launched lead to lasting changes in practice [23,24,25]. As one of the key factors of the reform was the transfer of responsibility from specialist to primary healthcare, coordination of patient care transitions is vital.

### Functional and normative integration

This study aims to investigate why care transitions continue to be challenging in spite of the new regime. By exploring how transitions between specialist and primary care are experienced by municipal actors, we will identify factors of organisational and professional integration between specialist and primary healthcare by applying elements of the Rainbow Model of Valentijn et al. [26]. Integration plays complementary roles on the macro, meso and micro levels. In contrast to other models [27,28,29], the concept of Rainbow Model emphasises functional and normative integration to ensure connectivity between the various levels. Functional integration is defined as key support functions and activities (i.e. financial, management and information systems) structured around the primary process of service delivery, to coordinate and support accountability and decision making between organisations and professionals in order to add overall value to the system. Normative integration is defined as the development and maintenance of a common frame of reference (i.e. a shared mission, vision, values and culture) between organisations, professional groups and individuals [26].

In recent years, a number of studies have used the Rainbow Model, or elements from this model, as an analytical and theoretical concept, see the examples of Angus & Valentijn [30], Breton et al. [31], Karlsson et al. [32], Nurjono & Shrestha [33] and van Rensburg & Fourie [34]. As far as we know, there are no empirical studies highlighting the meaning of functional and normative integration in care transitions for people with dual diagnosis seen from a municipal perspective. There is a need for increased attention to how system integration strategies interact with integration at the other levels [30]. We therefore explore care transitions in light of the policy framework of the Coordination Reform as experienced at the primary care level.

## Setting

The Coordination Reform contains a range of measures regarding patient transitions between primary care and hospitals (specialist care). Transitions are regulated by the Specialist Health Services Act, Municipal Health and Care Services Act and mutually binding cooperation agreements between the actors. The purpose of these agreements is to ensure a clear and efficient distribution of tasks and responsibilities and to create predictability in planning and budgeting the work for which each party is responsible. Guidelines have also been drawn up regarding measures to be cooperated on (Section 3-4 of the Health and Care Services Act and Section 2–1 of the Specialist Health Services Act). Here, the goal is to ensure that patients experience optimal coordination in healthcare and to prevent errors. The guideline also aims to ensure efficient, correct and secure information flow between and within the levels of healthcare. A written referral forms the basis for hospital admissions. The municipalities are allowed to refer patients to sociomedical polyclinics (SMP) but referrals to a district psychiatric centres (DPS) must be made by the patient’s GP. As for discharge from hospital, the definition of a patient ready for discharge is when the hospital doctor considers that no further treatment is needed in the specialist health service (Hospital website, 2017).

The healthcare system is divided into two separate governmental levels. In specialist care, SMP and DPS have regional outpatient and inpatient units for people with substance abuse problems and mental health problems, respectively. At the primary care level, municipalities as self-governing authorities are responsible for providing community health and social care along with GPs. All these actors play a role in patient care transitions between specialist and primary services.

## Methods

The research design of this case study is based on the research question of how patient transitions between primary and specialist health services were experienced by managers and front-line professionals. We used an explorative approach based on the methodological principles of qualitative case study research carried out in three municipalities [35,36]. The case was care transitions for people with dual diagnosis and the units were three municipalities, which varied in size, organisation of services and service complexity.

The participants were managers and front-line professionals in three municipalities in southern Norway. Municipality One is a small rural municipality (3,600 inhabitants). Their services for people with dual diagnosis are organised in a department with a unit for dual diagnosis that cooperates with other primary care services. Municipality Two is of medium size (31,200 inhabitants) and has one unit for mental health and substance use, led by a unit manager. There is a mental health team and a substance use team, each headed by a department manager. Municipality Three (about 50,000 inhabitants) is a large urban municipality, with a complex organisational structure.

In collaboration with researchers in a project, all authors conducted seven semi-structured group interviews in autumn 2018 during a full-day workshop. Two researchers headed each two-hour interview. The aim was to gain knowledge of how the managers and front-line professionals in each municipality experienced the services they provided to people with concurrent addiction and mental health problems. The agencies and organisations themselves and their managers selected participants for the workshop. The selection criterion was that front-line professionals from both mental health services and addiction services were represented. The group interviews included 11 front-line professionals and four managers. One manager and four professionals worked in Municipality One, and one manager and three professionals in Municipality Two. Two managers and four professionals worked in Municipality Three, see ***Table 1***.

**Table 1:** The different groups, topics and reflections.


**Municipal interviews, front-line professionals** Municipality One Municipality Two Municipality Three	**Mixed interview, managers**

**Theme:** Cooperation with specialist health services, especially the regional SMP and DPS units, and how they found cooperation with services at primary care level, such as GPs	**Theme:** Enablers and barriers for cooperation with specialist healthcare and the managers’ responses to governmental regulations.

**Mixed group interviews, front-line professionals** Group 1 Group 2 Group 3	

**Theme:** Issues across the three local services. How they experienced the services they provided to people with dual diagnosis.	


The workshop was divided into two sessions. We chose to use group interviews in both sessions, but with different compositions in order to elicit intra- and inter-municipal factors. All seven groups consisted of three to five participants. The managers from the three municipalities constituted one group. They all had a professional background and previous experience as front-line service providers. The objective of interviewing the managers in one group was to discuss some overriding issues across the three local services. Furthermore, in the first session all front-line professionals were divided into three groups according to the municipality for which they worked. The groups would thus be distinct from one another but the group members had a common work context to enable us to make comparisons across groups, here municipalities [37]. Some of the themes of the interview guide used here coincided with those used when interviewing the managers.

In the second session, the front-line professionals were again divided into three groups, but this time the groups were heterogeneous, having only one or two members from each municipality. This combination enabled the participants to exchange views across agencies and organisations and thus create different group dynamics from the previous group interview [38] (***Table 1***).

All interviews were audio-recorded and transcribed. The analytical approach was based on qualitative content analysis. First, all three authors read the interview material and the first and second authors developed codes, using an inductive data-driven approach [39]. Then, all three authors adopted a more deductive approach and drew on the analytical concepts of Valentijn et al. [26], regarding functional and normative integration.

In step one we concentrated on statements demonstrating the perspectives of local actors on patients’ care transitions. In step two, we identified and sorted meaning units relevant to the purpose of the study such as “challenges in use of e-link”. In the third step, the units were organised and coded into groups such as “challenges in communication and loss of meeting points”. In step four, we took a more deductive approach, drawing on the analytical concepts of Valentijn et al. [26], where we synthesised and condensed the content of each code group based on factors of organisational and professional integration. Finally, we contracted the condensed text into an analysis that constitutes our results [39]. These were refined in four main themes mainly regarding functional integration (***Table 2***).

**Table 2:** Codes and themes.


CODES	THEMES	FUNCTIONAL/NORMATIVE

GPs play a key role in transitions. Cooperation mostly by phone. GPs have time limits. Appointments are time-consuming.	Limited cooperation with GPs on referrals to hospital	Functional

Questioning GPs’ knowledge of dual diagnosis patients		Normative

Challenges in use of e-link. E-link is important but not integrated into patient record systems. Late or no notification of patient discharges. Previous collaboration meetings with hospital are missed.	Challenges of communication and loss of meeting points	Functional

SMP flexible and accessible. Allowed to refer to SMP. Close cooperation. Less cooperation with DPS. Not allowed to refer to DPS.	Close cooperation with the SMP, challenges in cooperation with the DPS	Normative Functional

Frustration at reduction in hospital beds. Have to relate to “untreatable” patients. Insufficient resources and facilities for providing new and advanced treatment.	Questioning hospital discharge of patients to primary care	Functional


The interpretation of the data was critically discussed with our colleagues in the research group throughout the analysis process. They all had read the material and could therefore support our interpretations, or put them to the test.

### Ethics and consent

NSD Data Protection Services approved the study, approval reference number 60390. The participants received both verbal and written information about the study. They were also informed that participation was voluntary and that they could withdraw from the study at any time. In addition, they received information about anonymisation and secure storage of the data.

## Results

We now present the findings of why care transitions are still tricky from the municipal perspective.

### Limited cooperation with GPs in referrals to hospital

GPs play a key role in care transitions and the municipal actors needed cooperation with patients’ GPs in order to support optimal coordination in the transitions. However, several participants conveyed experiences of limited cooperation with GPs around referrals to hospital admission. Cooperation mostly takes place by telephone, but they found this time-consuming as the GP was generally busy with other patients. Sometimes they needed to assist GPs in cases where patients needed an assessment from the GP for referral to hospital. They might be fortunate enough to get an emergency appointment and had to drive the patient to the GP’s surgery at that exact time, but then they had to wait in a waiting room with a sick patient, often spending the whole day there. However, there was always a risk that the patient might just get up and leave the surgery. Several participants felt that the doctor did not quite understand the patient’s problems and they found cooperation to be demanding. A further problem mentioned by the participants was that GPs had a time limit for appointments, which could result in exclusion of the patient. One service provider gave an example:

“So they allow you a quarter of an hour, like, and it takes a lot longer than that to get into detail with the patients we bring. So what happens is that they almost talk more to the care worker than to the patient. Yes, it’s quite a challenge.”

When front-line professionals had meetings with GPs, they often had to adapt to the doctor’s hours and then it could take a long time to arrange each meeting. GPs therefore sometimes did not attend the meetings. The participants pointed out that this depended on the GP’s priorities and the degree of severity of the patient’s condition. Not only was the time aspect highlighted with regard to GPs. Several of the participants questioned GPs’ knowledge and skills in dealing with dual diagnosis patients and stated that these doctors had difficulties in assessing them.

### Challenges of communication and loss of meeting points

The Coordination Reform highlights ICT as a significant means of improving communication and E-Link is an important communication channel between primary healthcare and medical services/emergency wards and hospitals for referrals and discharge summaries. This form of communication was, however, reported to be challenging. Furthermore, hospitals, GPs and municipal healthcare had different patient record systems that were not integrated. The participants emphasised the challenges involved in electronic communication on admissions and discharges. One of the front-line professionals stated that the municipality had an emergency admission and she wrote a good and clear admission report and asked for an urgent meeting with the hospital. This was done on a Friday, and the staff from the hospital ward called on the Saturday to ask if this patient had any primary follow-up care and hence revealed that they had not read the admission report:

“So is this a tool used for coordination, or is it just about making sure that … like ‘Check, check, check’, but is it used? Because if we can’t be sure that it’s being used, we can’t be sure of anything.”

This service provider reported that they had to make phone calls in addition to E-link and the other participants in the interview nodded when she said she was not sure if she could trust the electronic communication system. One said:

“I can’t call that message exchange system cooperation.”

The participants’ experiences of communication with specialist health services about discharges appeared to vary between the municipalities. In two municipalities, there seemed to be a general perception that they were not notified when a patient was ready for discharge, or even when the discharge was actually taking place. One of the front-line professionals had a flashback talking about being invited to meetings with the hospital before people were discharged in order to ensure agreement on the patient’s transition. He stated that those meetings hardly ever took place now:

“We have to ask time and again for a meeting before the person is discharged. But before we can blink an eye, we find the person is going home.”

Participants reported that late notifications or none at all might result in reduced opportunities for preventing errors in service delivery and poor planning for organising local services. In general, the participants said that they wanted regular collaboration meetings about patients with the specialist health services.

### Close cooperation with the SMP, challenges in cooperation with the DPS

Cooperation with the SMP was described as positive, especially in terms of flexibility and accessibility, while cooperation with the DPS was found to be more challenging.

“In my experience, we’ve worked hard at cooperation with the SMP for example, and it works very smoothly. We can refer patients to the SMP ourselves.”

Municipalities are allowed to refer patients to an SMP, but neither SMPs nor municipalities are allowed to refer to a DPS.

One front-line professional reflected on the cooperation they had with the SMP. They had stable staff turnover in the municipality as well as in the SMP. Over the years they had developed close cooperation and there was considerable contact between each individual substance abuse consultant in the municipality and each therapist in the SMP. The SMP therapists visited patients in their homes, preferably with someone from primary care. In this way, the SMP clinic appeared to be flexible from the professional’s point of view, but they also seemed to share a common understanding of a treatment repertoire for persons with substance abuse. With the DPS on the other hand, front-line professionals had less cooperation:

“I find the DPS to be much more rigid. The staff in the outpatient clinic don’t visit patients in their homes, and substance abuse can’t refer people to the SMP … Or the DPS, then GPs have to be involved.”

This statement reflects a loss of organisational structure for collaboration with the DPS. One manager expressed the challenges in getting dual diagnosis patients into a DPS ward because these wards are mainly oriented towards care and treatment for patients with mental disorders. For example, the DPS only provides treatment when a patient is intoxicated. This demonstrates the different organisational requirements for the SMP and DPS to collaborate with municipalities.

### Questioning hospital discharge of patients to primary care

The shift represented by the new regime seemed to frustrate the participants, as the following professional sighed:

“I don’t know what’s happened to all this slightly longer treatment for people with mental health problems then, even if they have a substance abuse problem too. Where are the wards, what happened to them? Where did they go? It’s kind of in and out.”

Both managers and front-line professionals identified major challenges related to the definition of the term “ready for discharge” and to the transfer of discharged patients back to primary care. Many patients have complex multimorbidities, they have nowhere to live when discharged, they do not easily “settle down” and municipal housing is either unsuitable or unavailable. In such cases, the participants reported disagreement about when a patient is ready for discharge and municipal care staff felt that their opinions carried little weight, as one stated:

“So then we have to come up with what we’ve got. So we think differently about what should be in place afterwards.”

The term “untreatable patients” was used during the interviews. Here, the participants questioned the guidelines for discharging patients from hospital. One of them stated:

“Yes, if they’re kind of ‘untreatable’, or whatever they call them in specialist healthcare, then it’s obvious we can’t discharge anyone. The social services are the lowest safety net in society - we have to look after everyone.”

Another front-line professional echoed this statement with the story of a patient who had been to a detoxification facility. Here, they were told by the hospital that the patient would be discharged on a certain date. However, the municipality did not have sufficient resources and facilities since the patient could not take care of himself, as the professional explained:

“…there were no vacancies, but we still get the message back: ‘The patient will be discharged on such and such a date’. And then it will be kind of… Well, we found a solution, but…”

One participant talked about a patient who was discharged because the hospital decided her treatment had finished. He elaborated on the disagreement about who should treat the patient:

“So because she had been out and in, and then it’s, it can be a quick discharge, and then she’s back in again, and it may have something to do with the Coordination Reform, that it should go to the municipality, so we just have to look after her.”

This participant put words to a key element of the Coordination reform.

## Discussion

By exploring how transitions between primary and specialist health services are experienced from a municipal perspective, we have identified factors of organisational and professional integration in patient care transitions between the two levels. Front-line professionals had limited cooperation with GPs in referrals to hospital and there were challenges in communication and meeting points. Further, they experienced close cooperation with the SMP, but challenges in cooperation with the DPS. A recurring theme was hospital discharge of patients to primary care. The governing framework of the Coordination Reform can be characterised as a new regime with its transfer of responsibility from specialist to primary healthcare in order to support integrated services. The old regime is represented by a long tradition of fragmentation and arbitrariness in healthcare, where the specialist health services were the focal point of treatment.

In line with the definition of Valentijn et al. [26], the Coordination Reform and subsequent legislation can be seen as policy instruments intended to create functional integration. Formal written agreements between hospitals and municipalities include guidelines for admissions and discharges. These guidelines are national standards to support integrated patient transitions at the organisational and professional level. The common guideline on patient discharge regulates how hospitals and municipalities can ensure that they agree on the definition of patients ready for discharge in line with the Coordination Reform. However, by questioning hospital discharge of patients to primary care, the municipalities seemed to act as if they retained the old model of hospital care (the old regime). Here, challenges can arise when the “traditional” professional roles and well-established routines meet new standards, but without the old roles being invalidated [40,41]. Municipal leaders and front-line professionals have to deal with a “double reality” where they will have to adapt to new practices represented by the Coordination Reform (the new regime) while also retaining the old model of hospital care (the old regime). Primary care did not seem to have sufficient resources and facilities for providing new and advanced treatment. Here we see that organisational interests and resources [42] are a key factor in integration. Thus, in terms of functional integration [26], use of the guideline for care transitions depended on organisational resources in order to work as a mechanism for integration. The participants reported challenges in cooperation with the DPS that can be explained by shortcomings in the system, namely the lack of access to treatment, the lack of integration between different specialist services, and coordination tools that are inadequate to prevent fragmented care pathways [9]. Studies of collaboration between DPSs and municipal mental health services also indicate that the structural framework for the DPS and local services overlaps and is incomplete [43]. Creating an organisational framework with a structure for meetings for learning, knowledge development and information exchange (normative integration) can enhance collaboration and help to coordinate services [44,45].

The various organisational and professional practices lead us to the importance of informal norms and values. As we see it, expressions of normative integration as defined by Valentijn et al. [26] can be linked to organisational culture as the most decisive factor in whether actors adopt the new policy framework or not [46,47]. The participants’ close cooperation with the SMP may be an expression of a common cultural professional norm between the participants and the SMP of how to relate to and treat people with dual disorders. The participants’ view of challenges in cooperation with the DPS may indicate that the SMP and DPS do not have the same organisational requirements for collaboration with municipal care providers. We see here an interdependence of functional and normative integration.

As long as the SMP and DPS are separate units in specialist health services, and primary care only has cooperation with one of the units, there is much to suggest that there is both functional and normative integration between primary care and the SMP but neither functional nor normative integration in the overall system of care transitions. There is a need for better understanding between the interacting parties, intra-level continuity of services, and more flexible procedures for patient transitions [43].

## Conclusion

We have applied the analytical framework of functional and normative integration in the Rainbow Model of Valentijn et al. [26] to explore why care transitions are still tricky after the introduction of the Coordination Reform in 2011. The policy framework represented by the Coordination Reform aimed at organisational and professional integration between specialist and primary care services. However, the new regime coexists with a long tradition of fragmentation in organisational structures and a divided professional culture that seems to result in a gap between visions of integration and actual practices. Based on our results, there is much to suggest the need for a greater focus on these requirements in order to fulfil the vision of integrated services.

The Rainbow Model of Valentijn et al. [26] is structured around primary care, with its focus on horizontal integration. In Norway, primary care needs close cooperation with specialist health services in order to meet patient needs. Thus, both horizontal and vertical integration are vital. The extent to which functional and normative integration can ensure connectivity between the different levels therefore depends on how services are organised.

## Limitations

The data collection was limited to managers and front-line professionals in three different municipalities representing experiences of primary care. We therefore have to take this into account, which implies a cautious interpretation of the findings. Additional interviews with GPs could have enhanced understanding of the primary care context. Likewise, interviews with actors in specialist health services could have provided a broader insight into the complexity of care transitions, involving the viewpoints of all implicated actors. However, highlighting the municipal perspective on factors that influence integration is important because the Coordination Reform has led to increased pressure on primary care. Our findings along with official reports and research on the Coordination Reform can enhance understanding of the organisational and professional complexity of integrated services.
